# Current management and future directions in the treatment of advanced renal cell carcinoma–a latin american perspective: 10 years in review

**DOI:** 10.1590/S1677-5538.IBJU.2014.0651

**Published:** 2015

**Authors:** Oren Smaletz

**Affiliations:** 1 Departamento de Oncologia, Hospital Israelita Albert Einstein, São Paulo, Brasil

**Keywords:** Kidney Neoplasms, Molecular Targeted Therapy, Angiogenesis Inhibitors, Vascular Endothelial Growth Factors

## Abstract

The worldwide incidence of kidney cancer is estimated at 337,860 new cases per year in the International Agency for Research on Cancer's GLOBOCAN 2012 update, with an estimated 143,369 deaths annually. Over the past 10 years, there have been significant advances in the treatment of advanced/metastatic renal cell carcinoma, including the development of targeted therapies. Currently recommended first-line treatments include sunitinib, temsirolimus, bevacizumab plus interferon, and pazopanib, or high-dose interleukin-2 or sorafenib for selected patients. Recommended second-line treatments include all of the above agents, as well as everolimus and axitinib. Unfortunately, combination therapies have generally resulted in increased toxicity and little improvement in efficacy. Recent studies focused on identification of predictive biomarkers for responses to specific targeted therapies and have not been successful to date. Despite recent advances in targeted treatment for metastatic renal cell carcinoma, important questions regarding biomarkers of efficacy, and optimal combination and sequencing of agents remain to be answered. This paper reviews literature concerned with first-and second-line treatment of metastatic renal cell carcinoma and will discuss key issues in Latin America.

## INTRODUCTION

Renal cell carcinoma (RCC) arises primarily from the proximal tubular epithelium and accounts for ~85% of all kidney cancers, with the remainder consisting of renal pelvis cancer and other rare malignancies (1). Many RCCs are asymptomatic and cannot be diagnosed until relatively late in the course of the disease. It has been estimated that more than 50% of RCCs are detected incidentally as a result of imaging tests carried out for other reasons, and that 25–30% of all patients with RCC are initially diagnosed due to symptoms of metastases (2, 3).

The age-standardized rates (ASRs) for kidney cancer incidence are similar in Latin America and the Caribbean (estimated ASR 3.5 per 100,000 population) to those in North America and Europe (ASR 3.6 and 3.3, respectively), while the ASR for mortality is slightly lower in Latin America and the Caribbean (estimated ASR 1.8 per 100,000 population) compared with that in North America and Europe (ASR 2.4 and 2.8, respectively) (4).

Due to the late stage at which many RCC patients are diagnosed, survival is often poor. The estimated average 5-year survival rate in the US is 91.7% for patients with localized disease, but only 12.3% for those diagnosed with distant metastases (5).

### Patient risk assessment and prognosis

In the cytokine treatment era, investigators at the Memorial Sloan-Kettering Cancer Center (MSKCC) developed a model for dividing patients with advanced disease in low-, intermediate-, and high-risk categories (6). Patients were assigned to one of three groups: those with zero risk factors (favorable risk), with one/two (intermediate risk), and with three or more (poor risk). Median overall survival (OS) for patients in these groups was 30, 14, and 5 months, respectively (6). Assessment of prognostic factors in patients with metastatic RCC (mRCC) treated with anti-vascular endothelial growth factor (VEGF) therapies led to a slightly different model (7), known as the International Metastatic Renal Cell Carcinoma Database Consortium model, in which neutrophilia and thrombocytosis are also considered independent prognostic factors, and has been recently validated; patients in the favorable, intermediate, and poor-risk groups had a median OS of 43.2, 22.5, and 7.8 months, respectively (8).

### Biomarkers

Despite considerable research, there are currently no validated biomarkers for use in the clinical management of mRCC, and only histology, staging, and clinical/laboratory characteristics can guide physicians in defining therapy and predicting patients’ outcomes. Nevertheless, biomarkers related to the VHL tumor suppressor gene, hypoxia-inducible factor (HIF), tumor-promoting genes responsive to HIF (e.g. those for VEGF, platelet-derived growth factor [PDGF], cyclin D1, glucose transporter 1), the mammalian target of rapamycin kinase (mTOR) pathway, the tumor suppressor gene phosphatase and tensin homolog, Akt, and phosphorylated S6K are all being explored (9). Single nucleotide polymorphism (SNP) genotyping is also being employed to identify significant polymorphisms in RCC-related genes related to prognosis; results to date suggest that polymorphisms in the interleukin (IL)-4 and VEGF genes are correlated with prognosis (9). A number of other biomarkers have shown prognostic value in clinical studies of targeted therapies for mRCC (10).

### Treatment of RCC

This review is focused on patients with advanced or mRCC. Stages I–III kidney cancers are managed with partial or radical nephrectomy, active surveillance or ablative techniques for non-surgical candidates (5). At this time, adjuvant strategies are not validated for the treatment of these stages.

### First-line treatment for advanced kidney cancer

**Figure 1:**
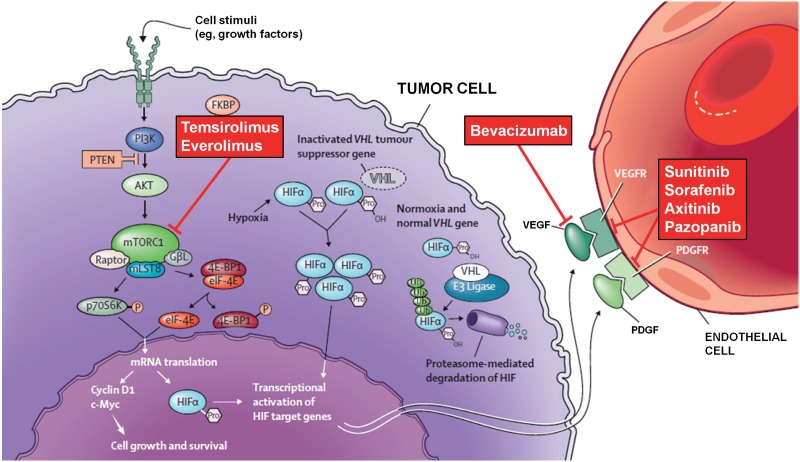
Therapeutic biological pathways for targeted therapies in mRCC (11).4E-BP1=4E binding protein-1; AKT=protein kinase B; FKBP=FK binding protein; eIF-4E=eukaryotic initiation factor-4 subunit E; FGF=fibroblast growth factor; HIF=hypoxia-inducible factor; IL-8=interleukin-8; mLST8=mammalian lethal with SEC13 protein 8; mTORC1=mammalian target of rapamycin complex 1; P70S6K=P70S6 kinase; PDGFR=platelet-derived growth factor receptor; P=phosphorous; PI3K=phosphoinositide 3-kinase; Pro=proline; PTEN=phosphatase and tensin homologue; Ub=Ubiquitin; VEGFR=vascular endothelial growth factor receptor; VHL=Von Hippel-Lindau.

Currently, the National Comprehensive Cancer Network (NCCN) guidelines’ recommended first-line treatments for metastatic and surgically unresectable RCC, supported by category 1 evidence, including sunitinib, temsirolimus (for poor-prognosis patients), bevacizumab plus IFN-α, and pazopanib (Figure 1; 5). Sunitinib and pazopanib are both tyrosine kinase inhibitors; targets of sunitinib include VEGF receptors (VEGFRs), PDGF receptors (PDGFRs), cKIT, and other kinases, while pazopanib also inhibits VEGFR, PDGFR, and cKIT. Bevacizumab is a monoclonal antibody that inhibits VEGF, and temsirolimus is an inhibitor of mTOR. With these agents, median progression-free survival (PFS) ranged from approximately 9 to 11 months in phase III studies, and median OS from 23 to 26 months; both parameters were shorter with temsirolimus, which was investigated in primarily poor-risk patients expected to have shorter survival (Table-1) (12–18).

This figure has been reproduced and modified with kind permission of Elsevier from Rini and Atkins. (Lancet Oncol, 2009) [permission to be obtained upon acceptance].

**Table 1:** Efficacy results from phase III studies with NCCN recommended first-line therapies for advanced RCC.

Test agent	Comparator	Progression-free survival				Overall survival			
		Median (months)		HR (95% CI)	p-value	Median (months)		HR (95% CI)	p-value
		Test	Comparator			Test	Comparator		
Sunitinib (12)	IFN-α	11.0	5.0	0.539 (0.451-0.643)	<0.001	26.4	21.8	0.821 (0.673-1.001)	0.051
Bevacizumab+IFN-α (13, 14)	IFN-α+placebo	10.2	5.4	0.63 (0.52-0.75)	0.0001	23.3	21.3	0.91 (0.76-1.10)	0.3360
Pazopanib (15, 16)	Placebo	9.2	4.2	0.46 (0.34-0.62)	<0.0001	22.9	20.5*	0.91 (0.71-1.16)	0.224
Pazopanib (17)	Sunitinib	8.4	9.5	1.05 (0.90-1.22)	NR	28.4†	29.3†	0.91 (0.76-1.08)	0.28
Temsirolimus (18)	IFN-α	5.5	3.1	NR	NR	10.9	7.3	0.73 (0.58-0.92)	0.008

* Overall survival analysis confounded by the early, high rate (54%) of crossover to placebo from pazopanib.

† Interim analysis of overall survival.

**CI=**confidence interval; **HR=** hazard ratio; **IFN-α=** interferon-α; **NCCN=** National Comprehensive Cancer Network; **NR=** not reported; **RCC=** renal cell carcinoma.

Despite differences in mechanism of action, the safety profiles of all four treatments share some similarities, with asthenia/fatigue (20-63%), nausea (26-52%), diarrhea (20-63%), and anorexia (22-37%) among the most commonly reported adverse events (AEs) (12, 13, 15, 17, 18). Hematologic toxicities, including leukopenia (37-78%), neutropenia (34-77%), lymphopenia (31-68%), and thrombocytopenia (32-78%), are also common with sunitinib and pazopanib. More unusual AEs include hand-foot syndrome (29-50%) and hypothyroidism (1424%) reported with sunitinib, bleeding events (33%) with bevacizumab, rash (47%) and pneumonitis with temsirolimus (19), and hypertension with all three VEGF inhibitors (any grade, 26-46%; grade 3/4, 3-15%). With each treatment, most AEs are mild to moderate (grade 1 or 2) and manageable with standard medical intervention or dosing modifications.

**Figure 2:**
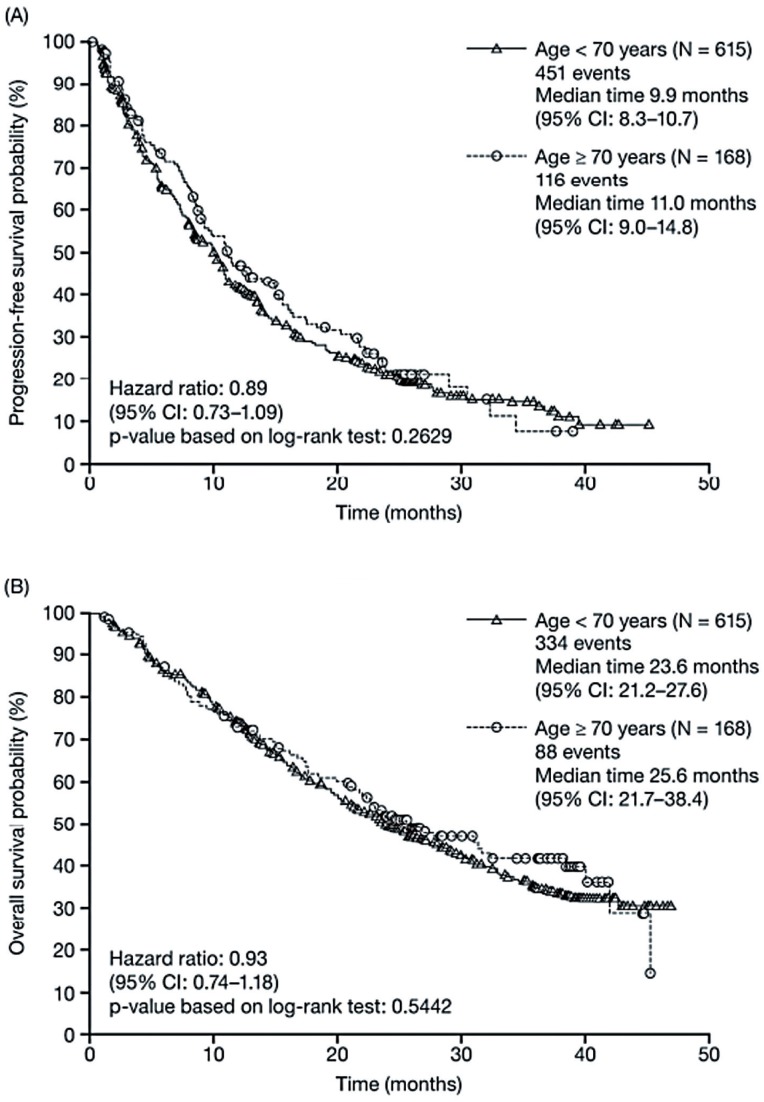
Progression-free survival (A) and overall survival (B) in sunitinib-treated patients by age (<70 vs. ≥70 years) in the first-line setting (23).

An expanded-access program was established to provide sunitinib to patients with mRCC who were ineligible for ongoing sunitinib clinical trials and/or before regulatory approval in their countries (20, 21). The program included 4564 patients from 246 sites in 52 countries, with 348 patients treated from Latin America. Overall efficacy and tolerability were similar among patients in this broader population to those participating in a phase III pivotal trial. Among Latin American patients, median PFS and OS were 12.1 and 16.9 months, respectively, 17% of patients had an objective response, and the clinical benefit rate (objective response plus stable disease≥3 months) was 57% (22). Responses were seen across all subgroups analyzed, including those with poor performance status, non-clear cell histology, or brain metastases (22). Results from a larger study found no significant effect of age on efficacy in patients receiving first-line or cytokine refractory sunitinib monotherapy for advanced RCC (20). For example, in first-line patients aged<70 and≥70 years, median PFS was 9.9 versus 11.0 months, respectively (hazard ratio (HR), 0.89; 95% confidence interval (CI): 0.731.09; p=0.2629) and median OS was 23.6 versus 25.6 months, respectively (HR, 0.93; 95% CI: 0.74-1.18; p=0.5442) (Figure-2) (23).

To date, there are few direct comparisons of the safety and efficacy of currently recommended first-line treatments for advanced RCC. The COMPARZ trial recently showed that pazopanib was non-inferior to sunitinib with respect to PFS, with an HR of 1.05 (95% CI: 0.90-1.22), and OS was similar (HR, 0.91; 95% CI: 0.76-1.08) (Table-1) (17). There were differences in the safety profile in patients treated with sunitinib compared with pazopanib, including a higher incidence of fatigue (63% vs. 55%, respectively), hand-foot syndrome (50% vs. 29%), and thrombocytopenia (78% vs. 41%), but a lower incidence of increased levels of alanine aminotransferase (43% vs. 60%). Similar proportions of patients needed dose interruptions or reductions because of toxicity, or discontinued treatment because of AEs. During the first 6 months of treatment, the mean change from baseline in 11 of 14 health-related quality of life domains favored pazopanib, particularly those related to fatigue or soreness in the mouth, throat, hands or feet (p<0.05 for all 11 comparisons).

There are still unanswered questions related to treatment selection for patients with advanced RCC (see below).

### Second-line treatment for advanced RCC

Current recommendations for second-line treatment of advanced RCC following a prior tyrosine kinase inhibitor include everolimus and axitinib, and following prior cytokine therapy include axitinib, sorafenib, sunitinib, and pazopanib (Figure-1; 5). The most recently approved of these agents, axitinib (approved in the US and Europe in 2012 and now also approved in several Latin American countries), is a selective and potent oral inhibitor of VEGFR-1,-2, and-3.

**Figure 3:**
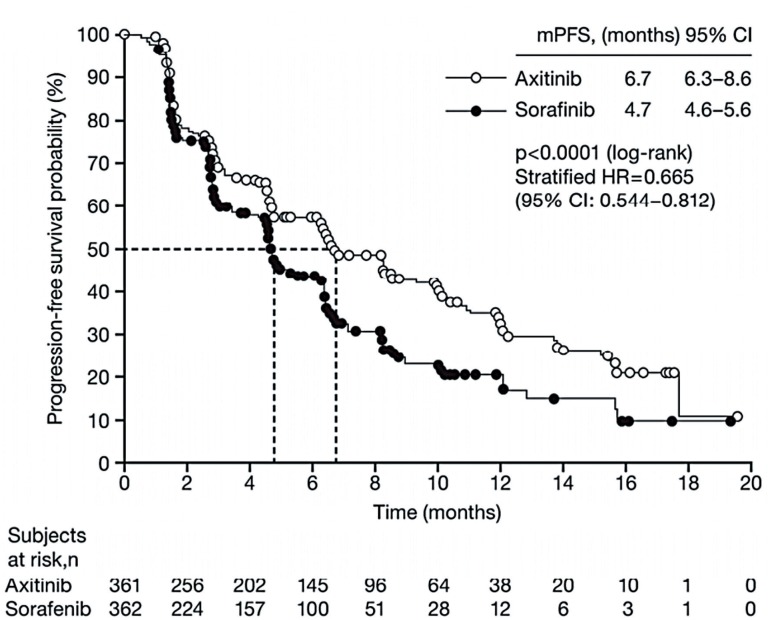
Progression-free survival with axitinib versus sorafenib as second-line therapy (24).

In a phase III study comparing axitinib with sorafenib as second-line treatment in 723 patients with clear-cell mRCC, median PFS was 6.7 months for axitinib and 4.7 months for sorafenib (p<0.0001) (Figure-3) (24) and the objective response rate (ORR) was 19.4% versus 9.4% (p=0.0001) (24). Updated results showed that OS did not differ between the two groups (median OS 20.1 months with axitinib vs 19.2 months with sorafenib; HR, 0.969; 95% CI: 0.8001.174; one-sided p=0.3744), but that investigator-assessed PFS remained longer with axitinib (median PFS 8.3 months) than with sorafenib (median PFS 5.7 months) (25). Common AEs occurring more frequently with axitinib than sorafenib were hypertension (40% vs. 29%, all grades), nausea (32% vs. 22%), dysphonia (31% vs. 14%), and hypothyroidism (19% vs. 8%); those occurring more frequently with sorafenib were hand-foot syndrome (27% vs. 51%), rash (13% vs. 32%), and alopecia (4% vs. 32%) (24).

This Figure has been reproduced with kind permission of Elsevier from Rini et al. (Lancet, 2011) [permission to be obtained upon acceptance].

To explore the efficacy of temsirolimus after VEGF inhibitor therapy, the INTORSECT trial compared temsirolimus with sorafenib as second-line treatment for patients with disease progression after sunitinib (26). PFS did not differ significantly between treatment arms (HR, 0.87; 95% CI: 0.71-1.07; two-sided p=0.19), but OS favored sorafenib (HR, 1.31; 95% CI: 1.05-1.63; two-sided p=0.01); median OS was 12.3 and 16.6 months in the temsirolimus and sorafenib arms, respectively. Safety data were as expected based on previous trials with each agent.

## COMBINATION TREATMENT

Many of the established and emerging treatments for mRCC have similar or overlapping biologic actions and we need more information about how they might influence each other's efficacy and the mechanisms underlying resistance to each (27). At present, it is unclear whether combination therapy aimed at vertical or horizontal inhibition is the best approach to second-line treatment. Vertical inhibition aims to block the same pathway at two points, as attempted by combining bevacizumab with sorafenib (28) or with sunitinib (29). These approaches were associated with improved activity, but also increased toxicity (28, 29). Horizontal inhibition combines agents of different mechanisms of action and non-overlapping toxicities with the goal of an additive or synergistic antitumor effect, as tested in the INTORACT trial which compared the combination of temsirolimus plus bevacizumab with interferon plus bevacizumab as first-line therapy in 791 patients with mRCC (30). Efficacy did not differ significantly between the treatment arms; median PFS was 9.1 and 9.3 months in the temsirolimus and interferon combinations arms, respectively (HR, 1.1; 95% CI: 0.9-1.3; p=0.8), and median OS was 25.8 and 25.5 months, respectively (HR, 1.0; p=0.6). Safety was consistent with the known profiles for the three agents. A recent randomized phase II trial in 361 treatment-naïve patients with advanced RCC compared single-agent bevacizumab with both vertical and horizontal combinations, namely temsirolimus plus bevacizumab, bevacizumab plus sorafenib, or sorafenib plus temsirolimus (31). However, none of the combinations tested were superior to single-agent bevacizumab with respect to PFS, and severe toxicity was increased with combination therapy. At this point, no combination has been shown to be superior to the approved combination of bevacizumab and interferon, and several combinations explored have been limited by excessive toxicity.

## CONTINUING EVOLUTION IN THE TREATMENT OF ADVANCED RCC

### Predicting treatment response

Identifying the optimum treatment for advanced RCC requires increased understanding of the tumor biology and patient characteristics predictive of response to specific treatments. A retrospective analysis based on pooled efficacy (n=544) and safety (n=4917) data from four clinical trials showed that sunitinib-induced hypertension was associated with significantly improved clinical outcomes (32). For patients with versus without hypertension, median PFS was 12.5 vs. 2.5 months, median OS was 30.9 vs. 7.2 months, and ORR was 54.8% vs. 8.7% (all p<0.001). In a similar study, using pooled data from 770 patients, patients who developed hand-foot syndrome had significantly better ORR (66.5% vs. 31.8%), median PFS (14.3 vs. 8.3 months), and median OS (38.2 vs. 18.9 months) than those not developing this AE (all p<0.0001) (33). Although confirmation in prospective studies is needed, one or more of these AEs may possibly serve as a predictive biomarker of sunitinib efficacy.

A gene expression profiling study identified a 20-gene signature predicting response to sunitinib with 68.5% accuracy (34), and microRNA profiling showed that 29 microRNAs were differentially expressed in patients with mRCC experiencing early progression on sunitinib (35). Analysis of potential soluble protein biomarkers found that lower angiopoietin-2 and higher matrix metalloproteinase-2 baseline levels were significantly associated with better overall response in patients treated with sunitinib, while higher tumor expression levels of HIF-1-α were associated with longer PFS (36). In patients treated with pazopanib, higher baseline plasma levels (relative to the median) of hepatocyte growth factor (HGF), IL-8, tissue inhibitor of metalloproteinases (TIMP)-1, and osteopontin were associated with shorter PFS (37). In the same study, high concentrations of IL-6 were predictive of improved PFS benefit from pazopanib compared with placebo. Single nucleotide polymorphisms in the genes IL8, FGFR2, NR1I2, and ABCB1 have all been associated with OS in patients with advanced RCC receiving pazopanib monotherapy (38).

## THE LATIN AMERICAN PERSPECTIVE

One consideration when using targeted agents to treat Latin American patients with mRCC is their different racial/ethnic mix compared with patients from North America. Results from a survey of 508 patients with RCC in Brazil indicated that 78.9% of patients were white, 6.5% were black, and 14.0% were mixed race (39). In contrast, results from a survey of 27,304 patients with RCC in the United States showed that 69.2% were white, 7.0% were black, 18.1% were Hispanic, and 5.0% were Asian/Pacific Islander (40). These small differences may influence the distribution of prognostic biomarkers as well as treatment efficacy and safety. On the other hand, a subpopulation analysis indicated that the efficacy and safety profile of sunitinib in patients with mRCC from Latin America who participated in a global expanded access program was comparable to that observed in the entire population. For example, in the Latin American and total populations, median PFS was 12.1 and 9.4 months, respectively, and median OS was 16.9 and 18.7 months, respectively (21, 22).

Drug availability is one of the key points to improved survival in patients with mRCC, as has been shown by the impact of post-progression therapy on OS in the clinical trial setting. In the AVOREN phase III trial of bevacizumab plus IFN-α, 63% of patients in the control arm (i.e. those randomized to IFN-α only) received at least one post-protocol therapy, comprising either sunitinib or sorafenib in 37% of cases (14). In this arm, median OS was 21.3 months, which is considerably longer than the median OS of approximately 13 months assumed for a patient population treated with IFN-α (6) when the trial was designed. In addition, a post-hoc exploratory analysis showed that median OS in patients randomized to bevacizumab plus IFN-α who received post-study tyro-sine kinase inhibitors was 38.6 months, compared with a median OS of 23.3 months in the same treatment arm of the intent-to-treat population (14). The CALGB phase III study of bevacizumab plus IFN-α also showed that median OS was greater for patients receiving further treatment after stopping trial therapy than for those receiving no subsequent therapy, regardless of treatment arm (28.2 months vs. 10.2 months) (41); however, in both arms patients receiving subsequent therapy had more favorable baseline prognostic features than those who were untreated.

These data show the positive impact of targeted therapy on survival in patients with advanced RCC. Nonetheless, with the exception of temsirolimus in poor risk patients, median OS, unlike PFS, was not significantly increased with targeted therapies compared with standard therapies or placebo in the phase III trials of first-line therapy (Table-1); however, as noted above, this is potentially due to post-protocol therapy received by patients in both treatment arms and, as discussed elsewhere (42), the confounding effect of crossover trial design. In addition, in some cases, postponing treatment with targeted therapy until a patient shows signs of disease progression may be prudent, due to the side effects associated with these treatments, which can potentially impact quality of life. Finally, these agents (and in particular sorafenib, sunitinib, and bevacizumab) are generally widely approved in Latin America (Table-2). However, regulatory approval does not guarantee widespread use of a drug. Clinicians from Latin American countries need to be more active in taking part in clinical trials of new drugs, which is an effective way of providing patient access to these agents, and also of making their benefits known to a wider population.

**Table 2:** Approval of targeted therapies by country in Latin America.

	Therapy							
Country	Sorafenib	Sunitinib	Pazopanib	Temsirolimus	Everolimus	Bevacizumab†	Axitinib	Observations
Argentina	√	√	√	√	√	√	√	Broad indication (everolimus after TKI failure only)
Brazil	√	√	√	√	√	√		Sorafenib second-line after cytokines; everolimus second-line after VEGFR TKI-based treatment
Chile	√	√	√	√	√	√	√	
Colombia	√	√	√	√	√	√	√	Sorafenib second-line after cytokines; everolimus second-line after VEGFR TKI-based treatment; temsirolimus in poor-prognosis patients
Venezuela	√	√	√	√	√	√		Broad indication
Ecuador	√	√	√			√	√	Broad indication
Peru	√	√	√			√	√	Broad indication
Mexico	√	√	√	√	√	√	√	Broad indication (everolimus after TKI failure only; pazopanib awaiting final approval)
Central-American countries*	√	√	√		√	√	√	Sorafenib second-line; sunitinib frst- or second-line; everolimus second-line after TKI failure

* Panama, Costa Rica, Nicaragua, El Salvador, Guatemala, Honduras, and Dominican Republic.

† Approved in combination with interferon-alpha.

**TKI=**tyrosine kinase inhibitor; **VEGFR=**vascular endothelial growth factor receptor.
